# Evaluation of the point-of-care Becton Dickinson Veritor™ Rapid influenza diagnostic test in Kenya, 2013–2014

**DOI:** 10.1186/s12879-016-2131-9

**Published:** 2017-01-11

**Authors:** Linus K. Ndegwa, Gideon Emukule, Timothy M. Uyeki, Eunice Mailu, Sandra S. Chaves, Marc-Alain Widdowson, Bandika V. Lewa, Francis K. Muiruri, Peter Omoth, Barry Fields, Joshua A. Mott

**Affiliations:** 1 DGHP, Centers for Disease Control and Prevention, Nairobi, Kenya; 2 Influenza Division, Centers for Disease Control and Prevention-Atlanta, Georgia, USA; 3 Kenya Medical Research Institute/Centers for Disease Control and Prevention-Kenya, Nairobi, Kenya; 4 Coast County Referral Hospital, Nairobi, Kenya; 5 Nyeri County Referral Hospital, Nairobi, Kenya; 6 Ministry of Health, Nairobi, Kenya; 7 Infection Control African Network (ICAN), Infection prevention network-Kenya (IPNET-K), Mbagathi Road off Mbagathi way, Village Market, PO Box 606, 00621 Nairobi, Kenya

**Keywords:** Influenza, Rapid influenza test, Sensitivity and specificity, Kenya

## Abstract

**Background:**

We evaluated the performance of the Becton Dickinson Veritor™ System Flu A + B rapid influenza diagnostic test (RIDT) to detect influenza viruses in respiratory specimens from patients enrolled at five surveillance sites in Kenya, a tropical country where influenza seasonality is variable.

**Methods:**

Nasal swab (NS) and nasopharyngeal (NP)/oropharyngeal (OP) swabs were collected from patients with influenza like illness and/or severe acute respiratory infection. The sensitivity, specificity, positive predictive value (PPV) and negative predictive value (NPV) of the RIDT using NS specimens were evaluated against nasal swabs tested by real time reverse transcription polymerase chain reaction (rRT-PCR). The performance parameter results were expressed as 95% confidence intervals (CI) calculated using binomial exact methods, with *P* < 0.05 considered significant. Two-sample Z tests were used to test for differences in sample proportions. Analysis was performed using SAS software version 9.3.

**Results:**

From July 2013 to July 2014, 3,569 patients were recruited, of which 78.7% were aged <5 years. Overall, 14.4% of NS specimens were influenza-positive by RIDT. RIDT overall sensitivity was 77.1% (95% CI 72.8–81.0%) and specificity was 94.9% (95% CI 94.0–95.7%) compared to rRT-PCR using NS specimens. RIDT sensitivity for influenza A virus compared to rRT-PCR using NS specimens was 71.8% (95% CI 66.7–76.4%) and was significantly higher than for influenza B which was 43.8% (95% CI 33.8–54.2%). PPV ranged from 30%–80% depending on background prevalence of influenza.

**Conclusion:**

Although the variable seasonality of influenza in tropical Africa presents unique challenges, RIDTs may have a role in making influenza surveillance sustainable in more remote areas of Africa, where laboratory capacity is limited.

## Background

In many tropical countries, the capacity for influenza virus diagnostic testing in clinical settings is limited. Although many point-of-care rapid influenza diagnostic tests (RIDTs) have been evaluated in temperate settings [1, 2], little information is available on the performance of RIDTs in tropical areas. Compared to temperate developed countries [3], the seasonality of influenza viruses is less predictable in sub-Saharan Africa (SSA). The populations of SSA also tend to be younger [4, 5], with more variable healthcare access and utilization [6], and have a greater prevalence of some chronic co-infections [7, 8]. Influenza has a clear marked seasonality in temperate regions [9–11]. This is less the case in the tropics where influenza circulation may occur throughout the year. RIDTs have been shown to perform well during the high influenza activity [12, 13]. However there are fewer data pertaining to their performance in clinical settings in tropical Africa where it is possible that RIDTs could facilitate greater awareness of influenza; improve the practical relevance of influenza surveillance in locations where laboratory confirmation may take days to weeks; and possibly inform clinical management and infection prevention and control practices at the clinic or hospital level [14]. The Becton Dickinson (BD) Veritor™ System (Becton, Dickinson and Company, Franklin Lakes, New Jersey) RIDT is intended for use in clinical settings. We implemented this test to detect seasonal influenza virus infections in both outpatient and inpatients reporting to existing respiratory disease surveillance systems in Kenya. We compared the performance of the BD Veritor test to detect influenza A and B infections, against the performance of real time reverse transcription polymerase chain reaction (rRT-PCR) that is used in the surveillance system.

## Methods

### Setting

**Fig. 1:**
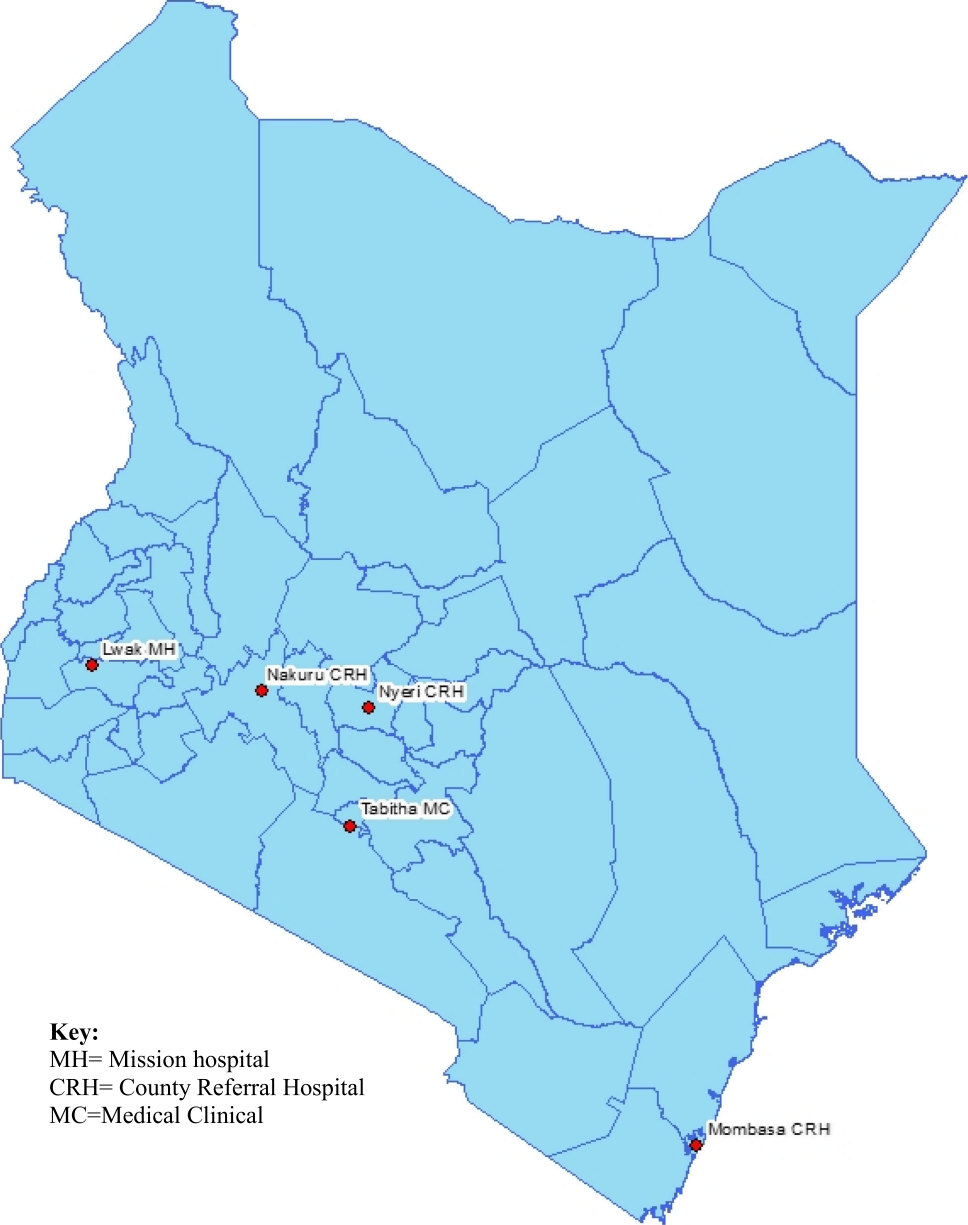
Map of Kenya showing the geographical location of the participating facilities

The study was implemented at five inpatient and outpatient influenza surveillance sites in Kenya. The five sites were selected to represent different geographical regions in Kenya (Fig. 1) and included: 1) Coast County referral hospital, located in the city of Mombasa; 2) Nakuru County referral Hospital, located in the Rift Valley; 3) Nyeri County referral Hospital, located in central Kenya; 4) St. Elizabeth Lwak Mission Hospital, a non-profit health facility in rural western Kenya that participates in the Population Based Infectious Disease Surveillance (PBIDS) system run by the Kenya Medical Research Institute (KEMRI) and the Centers for Disease Control (CDC) in Kenya, and; 5) Tabitha Medical Clinic, located in Kibera, an informal urban settlement in Nairobi [15, 16], that is also part of PBIDS. While Tabitha Medical Clinic is exclusively an outpatient clinic, all other facilities included inpatient and outpatient surveillance components.

### Study period

Influenza virus circulation in Kenya occurs year-round with primary peaks often occurring during the months of July–November, and secondary peaks during the period of March–May [4, 17]. For this study we recruited patients during a 13-month period from July 1, 2013 to July 31, 2014.

### Surveillance design and case definitions

Outpatient surveillance was conducted at each of the five sites for the first five daily cases of influenza like illness (ILI) identified among those aged ≥2 months. ILI was defined as axilla temperature ≥38 °C and either cough or sore throat with onset within the last seven days. Surveillance for severe acute respiratory illness (SARI) was undertaken at all five sites and captured all SARI cases identified throughout the day, although with some modification to the case definition applied. At three locations (Coast, Nyeri, and Nakuru County referral Hospitals), SARI was defined as history of fever or measured temperature ≥38 °C and cough, with onset within 14 days that required hospitalization. At the Lwak and Kibera clinics a modified “SARI” definition was used, where persons diagnosed with pneumonia [18] were considered a SARI case regardless of hospitalization status [19]. Therefore, in addition to presenting analysis based on ILI and SARI status we also present results by influenza activity periods, and inpatient vs. outpatient status, knowing that some outpatients may have had been classified as SARI due to pneumonia diagnosis without hospital admission. The low influenza periods were defined as <5% of tested ILI or SARI cases being laboratory confirmed as influenza during a specific month. Moderate influenza periods were those where 5–10% of ILI or SARI were laboratory confirmed; and high influenza periods were those where >10% of tests resulted in laboratory confirmed cases.

### Point of care testing

**Fig. 2:**
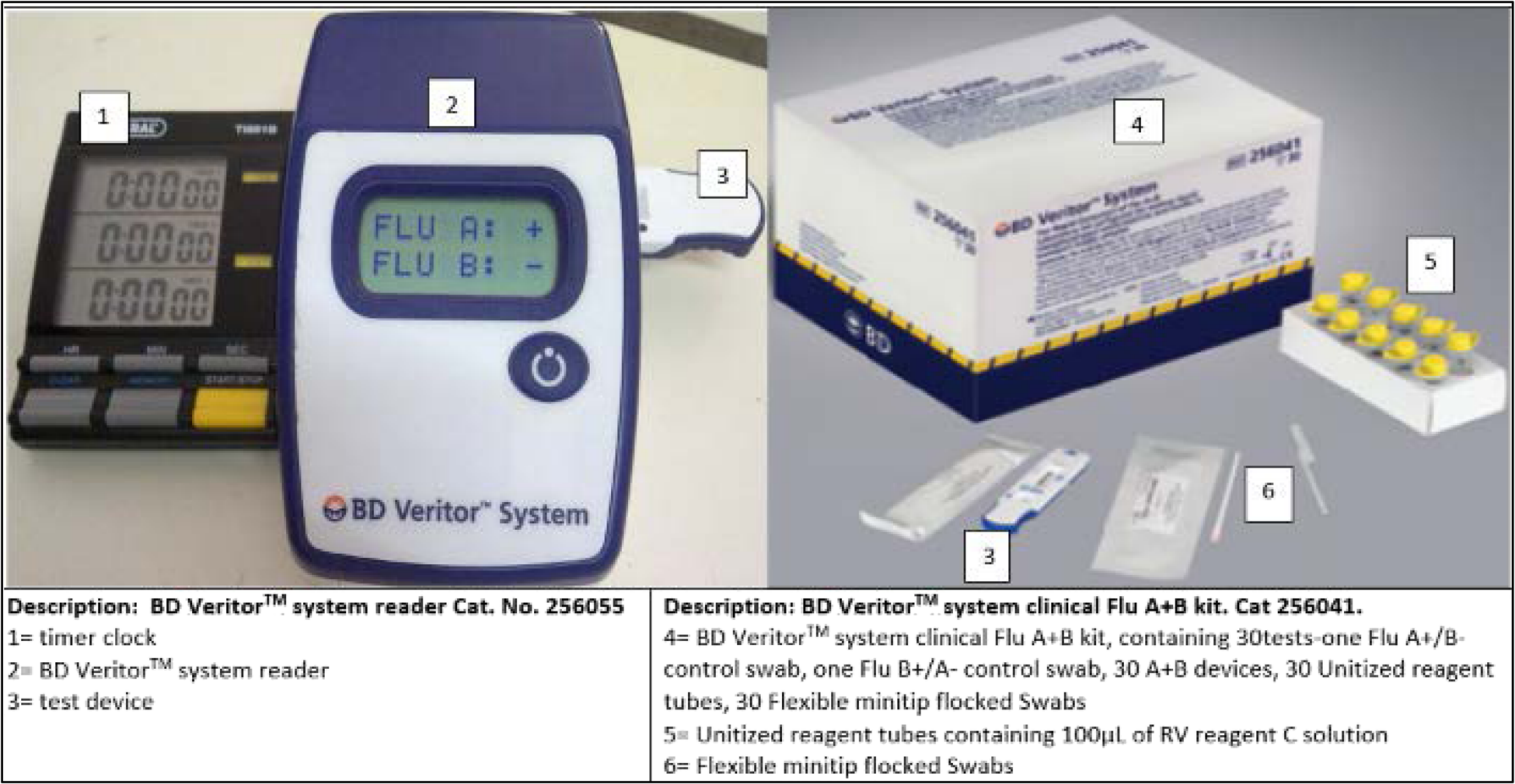
Description of BD Veritor^TM^ Rapid influenza diagnostic test kit

The BD Veritor™ RIDT is a rapid chromatographic immunoassay for the direct and qualitative recognition of influenza A and B viral nucleoprotein antigens in respiratory specimens (both from nasal and nasopharyngeal swabs) and produces results in ten minutes using an analyzer reader device [20] Fig. 2. The BD Veritor™ System for Rapid Detection of Flu A + B is a rapid chromatographic immunoassay for the direct and qualitative detection of influenza A and B viral nucleoprotein antigens from nasal and nasopharyngeal swabs of symptomatic patients. The BD Veritor System for Rapid Detection of Flu A + B (also referred to as the BD Veritor System and BD Veritor System Flu A + B) is a differentiated test, such that influenza A viral antigens can be distinguished from influenza B viral antigens from a single processed sample using a single device. The respiratory specimens are processed using BD RV Reagent D and then added to the test device. Influenza A or B viral antigens bind to anti-influenza antibodies conjugated to detector particles in the A + B test strip. The antigen-conjugate complex migrates across the test strip to the reaction area and is captured by the line of antibody on the membrane. A positive result for influenza A is determined by the BD Veritor System Reader when antigen-conjugate is deposited at the Test “A” position and the Control “C” position on the BD Veritor System Flu A + B assay device. A positive result for influenza B is determined by the BD Veritor System Reader when antigen-conjugate is deposited at the Test “B” position and the Control “C” position in the BD Veritor System Flu A + B assay device [20].

### Specimen collection and testing

Surveillance officers collected a nasal (NS) swab, a nasopharyngeal (NP) swab and an oropharyngeal (OP) swab from all patients who met the case definitions for ILI or SARI. All adult case-patients (aged ≥18 years) who met the ILI and SARI case definitions were asked to provide written informed consent. For all case-patients aged <18 years, written informed consent was provided by their parent/guardian. Children who were aged 7–17 years were considered mature minors and were asked to provide their assent in addition to their parent/guardian’s written informed consent.

One aliquot of a single NS specimen was tested on site using the BD RIDT, 2) the remaining aliquot from the same NS specimen was tested by real time reverse transcription polymerase chain reaction (rRT-PCR) at the KEMRI laboratory after being transported in a proprietary BD transport reagent. In addition, the NP and OP swabs were combined into a single vial of viral transport media according to Kenya’s routine surveillance standard operating procedures and tested at KEMRI laboratories using rRT-PCR.

All NS specimens were processed and tested by RIDT at each site according to the procedures in the BD RIDT package insert [20]. BD recommends nasal swabs or nasopharyngeal swabs specimens for testing with BD Veritor system Flu A + B test [20]. Briefly, a NS specimen was placed in a pre-labeled BD vial containing a proprietary BD transport reagent. The swab was then swirled in the media three times and three drops of the processed sample were then put on a labeled BD RIDT device and allowed to stand for exactly 10 min and then inserted into the BD Reader. The reader produces a positive or negative result for both influenza A and B viruses separately. After testing the NS samples at the site using BD RIDT, five drops of the remaining processed sample were added to BD Veritor system sterile proprietary BD transport media and kept at 4 °C in a refrigerator, before being shipped to the CDC-supported KEMRI laboratory in Nairobi, where they were stored at −70 °C awaiting processing by rRT-PCR.

Combined NP/OP samples collected as part of the ongoing influenza surveillance system [19] were placed in cryovials containing 1 mL sterile viral transport media (VTM) and kept at 4 °C in a refrigerator before being shipped to the KEMRI laboratory in Nairobi. All samples were transported from the sites to the laboratory within one week, maintaining cold chain throughout, where they were all tested for influenza viral ribonucleic acid (RNA) by rRT-PCR using specific primers and probes for influenza A and B viruses as previously described [4, 21].

RNA extraction from the respiratory samples was performed (after aliquots were done) using the viral RNA mini-kit (Quiagen, Germany) according to the manufacturer’s instructions; 140 μl of the respiratory sample in BD stabilizing reagent was added to a Lysis buffer solution for the cells to lyse and release the total RNA material. One step rRT-PCR was carried out using the AgPath kit (Applied Biosystems, California, USA). Following the reverse transcription step, a 45 cycle PCR reaction was run and fluorescence was read at the annealing/extension step. The primers, probes, and positive controls for all influenza viruses were provided by CDC-Atlanta. Appropriate negative and positive control specimens were run alongside each reaction. The results were recorded as cycle threshold (*C*_*T*_) values. When all controls met the stated requirements, any influenza A and B *C*_*T*_ value ≤ 39.9 was recorded as positive. Specimens with *C*_*T*_ values ≥ 40.0 were considered negative, and those without a C_T_ reading were recorded as negative.

We also documented the cost of purchasing the RIDT materials and cost for supplies and testing the samples using rRT-PCR, to calculate the cost of testing a sample using RIDT compared to rRT-PCR.

### Clinical and epidemiologic data collection

Clinical, demographic and epidemiological variables collected and analyzed included: sex and age of patient, SARI vs. ILI case status, site of testing, prevalence of influenza viruses during the month of testing, inpatient vs. outpatient case status, and days from symptom onset to specimen collection. These data, as well as the BD RIDT results, were recorded by participating clinicians on computers, smart phones or paper forms. Data collected on paper forms were entered into a Microsoft Access 2010 database (Microsoft, Redmond, WA, USA). Laboratory data were recorded into Freezer works software (Data works Development, Inc., 174, Mountlake Terrace, WA, USA) and merged with epidemiological information. All data were stored in Microsoft Access or Structured Query Language (SQL) databases on a central server at the CDC office on the KEMRI Campus in Nairobi.

### Statistical analysis

Analysis was performed using SAS software version 9.3 (SAS Institute, Cary, NC). Two evaluations of the BD RIDT were undertaken: 1) sensitivity, specificity, positive predictive value (PPV), and negative predictive value (NPV) of the RIDT were calculated compared to rRT-PCR performed on the same NS specimen stored in BD proprietary transport media; and 2) sensitivity, specificity, PPV, and NPV were estimated comparing BD RIDT results with rRT-PCR results from NP/OP samples placed in standard VTM, as is routinely undertaken for influenza surveillance in Kenya. The NS specimen tested at the KEMRI laboratories (i.e. comparison 1 above) was considered a “gold standard” for this analysis as this was the same specimen type that was used for testing by the RIDT.

All comparisons were performed within the following strata: sex (male, female), age (persons <5 years and persons aged ≥5 years), days from illness onset to sample collection (<2 days, ≥2–3, >3–4, >4–5, >5–6, >6–7, >7, and ≤7 days), days from sample collection to sample testing (≤7 vs. >7), days from illness onset to sample testing (≤7 vs. >7), inpatient vs. outpatient, influenza A vs. B virus, and by influenza prevalence (<5%, 5–10%, 10–15% and >15%). Influenza prevalence was based on monthly estimates of percentage influenza-positive NP/OP samples collected from ILI and SARI patients and tested by rRT-PCR during the study period. The performance parameter results were expressed as 95% confidence intervals (CI) calculated using binomial exact methods, with *P* < 0.05 considered significant. Two-sample Z test was used to test for differences in sample proportions.

## Results

**Fig. 3:**
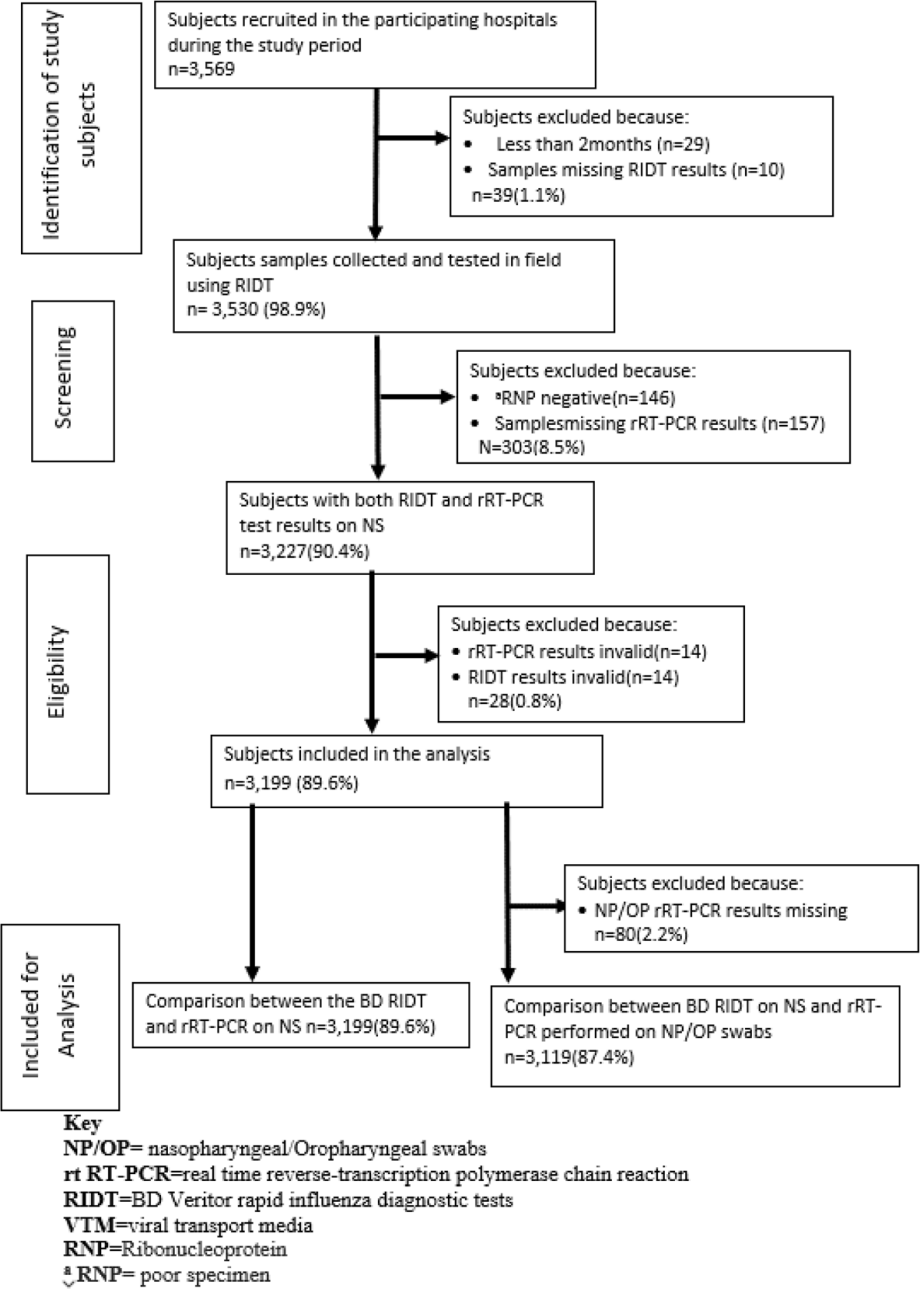
Study subjects flow diagram

During the study period (July 1, 2013 through July 31, 2014) 3,569 patients were enrolled (median age 2.1 years; range 2 months – 91 years). Of these, 3,199 patients provided NS specimens that were tested by BD RIDT and rRT-PCR; and 3,119 patients provided both NS and NP/OP specimens for testing by BD RIDT and rRT-PCR (Fig. 3). Patient characteristics are summarized in Table 1. The majority (78.5%) of the patients were aged <5 years. The median age and interquartile range for those aged <5 years was 1.4 years (0.72, 2.57) and for those ≥5 years was 10.5 years (6.6, 26.5). Overall, 27.3% (874 out of 3,199) of the patients presented to Nakuru, 22.6% (722 out of 3,199) to Kibera, 18.6% (595 out of 3,199) to Mombasa, 15.9% (509 out of 3,199) to Nyeri and 15.7% (501 out of 3,199) to Lwak. Overall, 64.4% of patients had SARI and 35.6% had ILI. Most (85.2%) patients had specimens collected ≤7 days after the onset of symptoms, of which 346 (12.7%) were collected in the first 48 h. The median time of illness of inpatients was 5.0 days and for outpatients was 3.0 days. Overall, 20% had specimens collected during low influenza periods 27.4% during moderate influenza periods and 52.6% during high influenza periods (Table 1).

**Table 1:** Demographic characteristic of the study participants in Kenya, July2013–July 2014 (*n* = 3,199)

Variable	Kibera *n* = 722		Lwak *n* = 499		Mombasa *n* = 595		Nakuru *n* = 874		Nyeri *n* = 509		Total	
Case Type		%		%		%		%		%	n	%
Department												
Outpatients	722	100.0	499	100.0	146	24.5	308	35.2	44	8.6	**1719**	53.7
inpatients	0	0.0	0	0.0	449	75.5	566	64.8	465	91.4	**1480**	46.3
Case Type												
ILI	350	48.5	292	58.5	146	24.5	308	35.2	44	8.6	**1140**	35.6
SARI	372	51.5	207	41.5	449	75.5	566	64.8	465	91.4	**2059**	64.4
Gender												
Female	373	51.7	251	50.3	240	40.3	407	46.6	208	40.9	**1479**	46.2
Male	349	48.3	248	49.7	355	59.7	467	53.4	301	59.1	**1720**	53.8
Age												
< 5 years	392	54.3	317	63.5	569	95.6	767	87.8	467	91.7	**2512**	78.5
≥ 5 years	330	45.7	182	36.5	26	4.4	107	12.2	42	8.3	**687**	21.5
Flu Activity												
Low < 5	121	16.8	90	18.0	109	18.3	130	14.9	190	37.3	**640**	20.0
Medium(≥5–10)	254	35.2	153	30.7	212	35.6	145	16.6	111	21.8	**875**	27.4
High(>10)	347	48.1	256	51.3	274	46.1	599	68.5	208	40.9	**1684**	52.6

*ILI influenza like illness*

*SARI Severe acute respiratory illness*

*In-Patients patients who had SARI and were exclusively admitted in the wards*

* if RNP negative it implies poor specimen

### Comparison of the BD RIDT to rRT-PCR performance on NS specimens (*N* = 3,199)

Of the respiratory specimens collected from 3,199 SARI and ILI patients tested using the BD RIDT, 462 (14.5%) were positive. Of these 462 RIDT positive specimens, 404 (87.4%) were positive for influenza A virus only, 56 (12.1%) were positive for influenza B virus only, and 2 (0.4%) were positive for both viruses. Of the 3,119 NS specimens tested using rRT-PCR, 417 (13.0%) were influenza virus positive. Of these, 417 influenza positive samples, 345 (82.7%) were positive for influenza A virus, 70 (16.8%) were positive for influenza B virus, and 2 (0.5%) were positive for both viruses. When compared to NS specimens placed in BD transport media and tested by rRT-PCR, the BD RIDT had an overall sensitivity to detect influenza A or B viruses of 77.0% (95% Cl, 72.6%–80.9%), a specificity of 94.9% (95% Cl, 94.0%–95.7%), PPV of 69.5% (95% Cl, 65.0%–73.6%), and NPV of 96.5% (95% Cl, 95.7%–97.1%) (Table 2). The PPV was 81.6% (95% Cl, 72.3%–88.5%) for outpatients and 65.9% (58.9%–72.7%) for inpatients.

**Table 2:** Performance of BD rapid test compared with rRT-PCR test for nasal swabs in Kenya, July 2013–July 2014 (*N* = 3,199)

Characteristic	Specimens		%Sensitivity	%Specificity	%PPV	%NPV
	n,	%positive	(CI, 95%)	(CI, 95%)	(CI, 95%)	(CI, 95%)
Influenza Type						
Influenza A or B	321	10.0	77.0 (72.6–80.9)	94.9 (94.0–95.7)	69.5 (65.0–73.6)	96.5 (95.7–97.1)
Influenza A	270	8.4	77.8 (73.0–82.0)	95.8 (94.4–96.0)	66.5 (61.7–71.0)	97.2 (96.5–97.8)
Influenza B	46	1.4	63.9 (51.7–74.6)	99.6 (99.3–99.8)	79.3 (66.3–88.4)	99.2 (98.9–99.4)
Case Type:						
SARI (*n* = 2059)	178	8.6	74.2 (68.1–79.5)	94.9 (93.7–95.8)	65.7 (59.7–71.3)	96.5 (95.6–97.3)
ILI (*n* = 1140)	143	12.5	80.8 (73.4–82.4)	95.0 (94.4–96.0)	74.9 (61.5–70.9)	96.4 (96.7–97.9)
Department:						
Inpatient^a^ (*n* = 1480)	122	8.2	76.3 (69.3–82.8)	95.2 (93.9–96.3)	65.9 (58.9–72.7)	97.1 (96.0–97.9)
Outpatient (*n* = 498)	80	16.1	87.0 (77.9–92.8)	95.6 (93.0–97.3)	81.6 (72.3–88.5)	97.0 (94.7–98.4)
Age:						
< 5 years (*n* = 2512)	237	9.4	78.0 (72.8–82.4)	95.1 (94.1–96.0)	68.7 (63.5–73.5)	96.9 (96.1–97.6)
≥ 5 years (*n* = 687)	84	12.2	74.3 (65.1–81.9)	94.3 (91.9–96.0)	71.8 (62.6–79.5)	94.9 (92.7–96.5)
Illness onset to sample collection:						
Less than 2 days (*n* = 346)	23	6.6	65.7 (47.7–80.3)	92.0 (88.2–94.6)	47.9 (33.5–62.6)	96.0 (92.9–97.8)
≥ 2 to 3 days (*n* = 1267)	145	11.4	79.2 (75.5–84.7)	95.5 (94.0–96.6)	74.7 (67.9–80.6)	96.5 (95.1–97.4)
> 3 to 4 days (*n* = 454)	44	9.7	80.0 (66.7–89.1)	94.5 (91.6–96.4)	66.7 (53.9–77.5)	97.2 (94.8–98.5)
> 4 to 5 days (*n* = 337)	30	8.9	75.0 (58.5–86.8)	96.0 (92.9–97.8)	71.4 (55.2–83.8)	96.6 (93.7–98.3)
> 5 to 6 days (*n* = 237)	21	8.9	77.8 (57.3–90.6)	95.2 (91.2–97.6)	67.7 (48.5–82.7)	97.1 (93.5–98.8)
> 6 to 7 days (*n* = 174)	18	10.3	69.2 (48.1–84.9)	93.9 (88.4–97.0)	66.7 (46.0–82.8)	94.6 (89.2–97.4)
> 7 days (*n* = 298)	23	7.7	76.7 (57.3–89.4)	95.9 (92.6–97.8)	67.6 (49.4–82.0)	97.3 (94.4–98.8)
≤ 7 days (*n* = 2727)	280	10.3	77.6 (72.8–81.7)	94.9 (93.9–95.7)	69.8 (65.0–74.2)	96.5 (95.7–97.2)
Sample collection to sample testing^b^:						
> 7 days (*n* = 2834)	305	10.8	76.3 (71.9–80.4)	95.2 (94.3–96.0)	72.4 (67.9–76.6)	96.1 (95.2–96.8)
≤ 7 days (*n* = 365)	16	4.4	94.1 (69.2–99.7)	92.8 (89.4–95.2)	39.0 (24.6–55.5)	99.7 (98.0–100)
Influenza Prevalence						
Influenza activity <5% (*n* = 640)	13	2.0	72.2 (46.4–89.3)	95.7 (93.7–97.1)	32.5 (19.1–49.2)	99.2 (98.0–99.7)
Influenza activity 5to10% (*n* = 875)	42	4.8	76.4 (62.7–86.3)	95.0 (93.2–96.3)	50.6 (39.5–61.7)	98.4 (97.2–99.1)
Influenza activity >10to15% (*n* = 354)	28	7.9	71.8 (54.9–84.5)	95.2 (92.1–97.2)	65.1 (49.0–78.5)	96.5 (93.6–98.1)
Influenza activity >15% (*n* = 1334)	238	17.9	78.1 (73.0–82.5)	94.4 (92.7–95.7)	80.5 (75.4–84.7)	93.5 (91.8–94.9)
Influenza activity ≥10%(*n* = 1684)	266	15.8	77.3 (72.5–81.6)	94.6 (93.2–95.7)	78.5 (73.6–82.6)	94.2 (92.8–95.4)
Flu activity <10% (*n* = 1515)	56	3.6	75.3 (63.6–84.4)	95.3 (94.0–96.3)	44.7 (35.8–53.9)	98.7 (97.9–99.2)
Surveillance Site:						
Kibera (*n* = 722)	85	11.8	73.9 (64.7–81.5)	95.6 (93.5–99.7)	75.9 (66.7–83.3)	95.1 (93.1–96.6)
Lwak (*n* = 499)	34	6.8	68.0 (53.2–80.1)	92.7 (89.7–94.8)	50.7 (38.4–63.0)	96.3 (94.0–97.8)
Mombasa (595)	55	9.2	79.7 (68.0–88.1)	95.1 (92.7–96.7)	67.9 (56.5–77.6)	97.3 (95.4–98.4)
Nakuru(874)	110	12.6	84.6 (77.0–90.1)	94.0 (91.9–95.5)	71.0 (63.0–77.8)	97.2 (95.7–98.3)
Nyeri (*n* = 509)	37	7.3	69.8 (55.5–81.3)	97.8 (95.9–98.9)	78.7 (63.9–88.8)	96.5 (94.3–97.9)

*ILI influenza like illness, SARI Severe acute respiratory illness*

^*a*^ *In-Patients patients who had SARI and were exclusively admitted in the wards*

^*b*^ *Time to sample tested refer to the rRT-PCR only since all RIDT was performed at bedside*

*PPV* positive predictive value

*NPV* negative predictive value

*CI Confidence interval*

Table 2, shows the performance of BD veritor^TM^ rapid test compared with real time reverse-transcription polymerase chain reaction (RT-PCR) test method for nasal swabs, by type of influenza, age, influenza prevalence, time of illness onset to sample collection and site in Kenya for the study period. The sensitivity and specificity of the BD RIDT did not significantly vary by case type, age, duration of illness onset to sample collection, influenza prevalence, virus type, or surveillance site (Table 2).

**Fig. 4:**
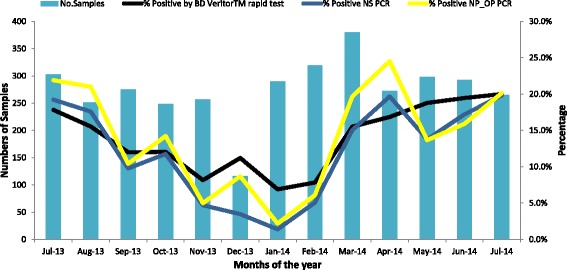
Comparison of influenza positive specimens by month using RIDT and rRT-PCR

Figure 4, shows comparison of influenza positive specimens by month using the BD Veritor^TM^ Rapid influenza diagnostic test, rRT-PCR performed on nasal specimens, and rRT-PCR performed on Nasopharyngeal/Oropharyngeal specimens, during the study period. During the study period, influenza virus detection demonstrated a marked seasonal variability, ranging from 2.2% to 21.4% (Fig. 2). The PPV of the BD RIDT was 78.5% (95% CI 73.6–82.6) for the 1,684 specimens collected during periods when influenza virus prevalence in SARI and ILI cases was ≥10%, which was significantly higher than the PPV of 44.7% (95% CI 35.8–53.9) for the 1,515 specimens collected during periods when influenza virus prevalence was <10% (*p* < 0.0001). Of the respiratory specimens from 3,119 SARI and ILI patients tested using BD RIDT, 452 (14.5%) were positive for an influenza virus, and of these influenza positive specimens, 394 (87.2%) were positive for influenza A virus only, 56 (12.4%) were positive for influenza B virus only, and 2 (0.4%) were positive for both viruses. Of the 3119 NP/OP specimens tested using rRT-PCR, 438 (14.0%) were influenza virus positive and of these, 343 (78.3%) were positive for influenza A virus, 92 (21.0%) were positive for influenza B virus, and 3 (0.7%) were positive for both influenza A and B viruses.

Table 3, shows the performance of BD veritor^TM^ rapid test compared with real time reverse-transcription polymerase chain reaction (RT-PCR) test method for nasopharyngeal/oropharyngeal swabs, by type of influenza, age, influenza prevalence, time of illness onset to sample collection and site in Kenya for the study period. The BD RIDT on NS specimens compared to rRT-PCR on NP/OP swabs had an overall sensitivity of 67.4% (95% C.I 63.0–71.7%); with a greater sensitivity to detect influenza A virus 71.7% (95% C.I 66.8–76.4) when compared to influenza B virus 43.2% (95% C.I 33.2–54.1%; *p* < 0.0001). When detecting influenza A or B virus the overall specificity was 94.1% (95% C.I 93.2–95.0%); the PPV was 65.3% (95% C.I 60.9–69.6%); and the NPV was 94.6% (95% C.I 93.8–95.5%). The sensitivity and specificity of the BD RIDT did not significantly vary by case type, inpatient or outpatient status, age, duration of illness onset to sample collection, influenza prevalence, or surveillance site (Table 3). However, as in the previous comparison, the PPV of the BD RIDT was significantly lower during periods when the prevalence of influenza viruses was <10% compared to when it was ≥10% (*p* < 0.0001). The PPV to detect influenza viruses was significantly higher in outpatient than inpatient settings (*p* < 0.0005) (Table 3).

**Table 3:** Performance of BD rapid test compared with rRT-PCR test for NP/OP swabs in Kenya, July 2013–June 2014 (*N* = 3,119)

Characteristic	Specimens		%Sensitivity	%Specificity	%PPV	%NPV
	n	%positive	(CI, 95%)	(CI, 95%)	(CI, 95%)	(CI, 95%)
Influenza Type						
Influenza A or B	295	9.5	67.4 (63.0–71.7)	94.1 (93.2–95.0)	65.3 (60.9–69.6)	94.6 (93.8–95.5)
Influenza A	248	8.0	71.7 (66.9–76.4)	94.7 (93.8–95.5)	62.6 (57.9–67.4)	96.4 (95.7–97.1)
Influenza B	41	1.3	43.2 (33.2–54.1)	99.4 (99.2–99.7)	70.7 (58.0–82.4)	98.2 (97.8–98.7)
Case Type:						
SARI (*n* = 1995)	160	8.0	65.0 (58.7–70.9)	94.1 (92.9–95.1)	60.8 (54.6–66.7))	95.0 (93.9–96.0)
ILI (*n* = 1124)	135	12.0	70.3 (63.2–75.6)	94.2 (92.5–95.6)	71.4 (63.3–77.6)	93.9 (92.1–95.3)
Department:						
Inpatient^a^ (*n* = 1,429)	105	7.3	69.5 (61.4–76.6)	94.1 (92.6–95.3)	58.0 (50.4–65.2)	96.3 (95.1–97.3)
Outpatient (*n* = 489)	77	15.7	80.2 (70.5–87.4)	94.7 (91.8–96.6)	78.6 (68.9–86.0)	95.1 (92.4–97.0)
Age:						
< 5 years (*n* = 2,443)	217	8.9	70.5 (65.0–75.4)	94.2 (93.1–95.1)	63.7 (58.4–68.9)	95.7 (94.9–96.6)
≥ 5 years (*n* = 676)	78	11.5	60.0 (51.0–68.4)	93.8 (90.5–94.9)	69.6 (57.3–74.9)	90.8 (88.1–93.1)
Duration of illness onset to sample collection:						
Less than 2 days (*n* = 341)	23	6.7	54.8 (38.8–69.8)	92.0 (88.1–94.7)	48.9 (34.3–69.7)	93.5 (90.0–96.0)
≥ 2 to 3 days (*n* = 1245)	133	10.7	68.2 (61.1–74.6)	94.5 (92.9–96.7)	69.6 (62.5–76.0)	94.1 (92.5–95.4)
> 3 to 4 days (*n* = 443)	44	9.9	69.8 (56.8–80.4)	94.2 (91.2–96.3)	66.7 (53.9–77.5)	95.0 (92.1–96.9)
> 4 to 5 days (*n* = 326)	25	7.7	73.5 (55.3–86.5)	94.5 (91.1–96.7)	61.0 (44.5–75.4)	96.8 (93.9–98.5)
> 5 to 6 days (*n* = 232)	21	9.1	75.0 (54.8–88.6)	95.1 (90.9–97.5)	67.7 (48.5–82.7)	96.5 (92.7–98.5)
> 6 to 7 days (*n* = 170)	16	9.4	59.3 (39.0–77.0)	93.0 (86.7–96.0)	61.5 (40.7–79.1)	92.4 (86.7–96.0)
> 7 days (*n* = 453)	34	7.5	61.8 (47.7–74.3)	93.7 (90.6–95.8)	56.6 (44.1–70.2)	94.7 (91.8–96.6)
≤ 7 days (*n* = 2666)	261	9.8	68.1 (63.2–72.7)	94.2 (93.2–95.1)	66.4 (61.5–71.0)	94.6 (93.6–95.5)
Time of sample collection to sample testing:						
> 7 days (*n* = 2,773)	281	10.1	67.7 (62.9–72.1)	94.4 (93.3–95.2)	67.9 (63.1–72.3)	94.3 (93.3–95.2)
< 7 days (*n* = 346)	14	4.0	60.9 (38.8–79.5)	92.6 (89.0–95.1)	36.8 (22.3–54.0)	97.1 (94.4–98.6)
Influenza Prevalence						
Flu activity <5% (*n* = 619)	11	1.8	91.7 (59.8–99.6)	95.6 (93.5–97.0)	28.9 (16.0–46.1)	99.8 (98.9–100)
Flu activity ≥ 5to < 10%(*n* = 857)	33	3.9	53.2 (40.2–65.8)	94.1 (92.3–95.6)	41.3 (30.5–52.8)	96.3 (94.6–97.4)
Flu activity >10–15% (*n* = 342)	28	8.2	70.0 (53.3–82.9)	95.0 (91.8–97.1)	65.1 (49.0–78.5)	96.0 (92.9–97.8)
Flu activity >15% (*n* = 1301)	223	17.1	68.8 (63.4–73.8)	93.0 (91.2–94.5)	76.6 (71.3–81.3)	90.0 (87.9–91.7)
Flu activity ≥10% (*n* = 1,643)	251	15.3	69.0 (63.9–75.6)	93.5 (92.0–94.8)	75.1 (70.1–79.6)	91.4 (89.7–92.8)
Flu activity <10% (1,476)	44	3.0	59.5 (47.4–70.5)	94.7 (93.4–95.8)	37.3 (28.7–46.7)	97.8 (96.9–98.5)
Site:						
Kibera (*n* = 707)	79	11.2	60.3 (51.4–68.6)	95.3 (93.2–96.8)	74.5 (65.0–82.3)	91.3 (88.7–93.4)
Lwak (494)	34	6.9	56.7 (43.3–69.2)	92.4 (89.5–94.7)	50.7 (38.4–63.0)	93.9 (91.1–95.9)
Mombasa(*n* = 562)	45	7.9	72.6 (59.6–82.8)	93.3 (90.6–95.2)	57.0 (45.4–67.9)	96.5 (94.4–97.9)
Nakuru (*n* = 851)	102	12.0	77.9 (69.6–84.4)	92.9 (90.7–94.6)	66.7 (58.5–73.9)	95.8 (94.0–97.2)
Nyeri (*n* = 500)	35	7.0	64.1 (50.6–77.8)	97.3 (95.2–98.5)	74.5 (59.4–84.9)	95.8 (93.4–97.4)

^a^n for inpatient/outpatient =1944; *ILI* influenza like illness, *SARI* severe acute respiratory illness

*In-Patients* patients who had SARI and were exclusively admitted in the wards

*PPV* positive predictive value

*NPV* negative predictive value

*CI* confidence interval

*rRT-PCR* real time reverse-transcription polymerase chain reaction

*NP/OP* nasopharyngeal/Oropharyngea

## Discussion

In this study we undertook a large point of care evaluation of the BD Veritor™ RIDT in clinical surveillance settings at multiple geographic localities in Kenya. When compared to NS specimens tested by rRT-PCR, the overall sensitivity of the BD RIDT to detect influenza A or B viruses was 77%, and specificity was 95%, similar to the performance of RIDTs in clinical care settings reported in temperate countries [22, 23]. Although RIDTs can never completely replace the need for viral culture to characterize influenza viruses, they may have a role to play in sub-Saharan Africa as they have the potential to reduce laboratory transport and diagnostic costs in routine influenza surveillance. RIDTs also have potential to inform clinicians and public health departments about when influenza viruses are circulating and relative levels of influenza activity.

The per-specimen rRT-PCR cost, including reagent purchases and RNA extractions at the KEMRI laboratory in Kenya (not including specimen collection and transport) is ~ $13.3 USD. This can conservatively be compared to a per-test cost of $10.6 USD of the BD-Veritor system. In the context of many competing priorities and funding challenges, the sustainability of influenza sentinel surveillance is a serious consideration in many African countries [21, 24, 25]. As a result, innovative methods to reduce the cost of surveillance and to generate timely feedback of test results to clinicians have to be considered, perhaps with a known subset of specimens (e.g. those of greater severity, those in severe illness clusters, or those with epidemiologic-linkages to high risk exposures etc.) sent for rRT-PCR and viral culture analyses.

The moderately high sensitivity observed in this study may relate to the fact that 80% of the ILI and SARI case-patients tested were aged <5 years, and RIDTs have been shown to have relative high sensitivity among young children because of increased influenza viral load compared to older children and adults [26, 27]. The sensitivity of RIDTs can be substantially diminished among adults (~18%), [28] and some studies have shown even lower sensitivity among those ≥65 years (8%) [29–31], leading to underestimation of disease burden [32, 33].

There were no significant differences in the sensitivity and specificity of the BD RIDT compared to rRT-PCR by age, surveillance site, and duration from illness onset to sample collection. However, as expected, PPV was substantially lower during low influenza periods [13, 34]. The PPV was ~30% for specimens tested by the RIDT during low influenza activity (<5% influenza-positive samples), and ~50% when the prevalence of positive samples for influenza was between 5–10%.

While RIDTs have some promise for surveillance purposes, the relatively unpredictable and variable seasonality of influenza in Kenya also presents some challenges to the practical utility of RIDTs [4, 35] for clinical decision-making. In the U.S., empiric antiviral treatment is recommended as soon as possible for patients with confirmed or suspected influenza who are at increased risk for complications from influenza, without waiting for the results of influenza testing. This is possible because negative test results (especially RIDTs) do not exclude a diagnosis of influenza during influenza season [36]. This problem would only be exacerbated in the Kenyan context where there is continuous but variable annual circulation. In low resource settings there may be a desire to base use of antiviral therapy on positive RIDT results; however, the high frequency of false positive results during periods when influenza prevalence is <10% would result in inappropriate use of antiviral treatment for many RIDT positive patients.

Our study had several limitations. Even though our intent was to evaluate the performance of the RIDT in patients of all ages, our study population was primarily children <5 years, with median age of 1.4 years, and therefore our findings cannot be generalized to all adults. Also, these results reflect a single year of influenza virus circulation in Kenya and could vary in other years when influenza activity has different patterns and levels. Although almost half of the cases presented within 72 h of illness onset, most samples (88.6%) were tested >7 days from the time they were collected, and this could have reduced the sensitivities of both the RIDT and rRT-PCR to detect influenza viruses. The prolonged time in the freezer, before testing, could have affected the sensitivity of the rRT-PCR, hence the lower rate of positivity by rRT-PCR when tested >7 days compared to RIDT. However, Caselton, et al. [37], there was no statistically significant difference in influenza positivity of specimens stored up to five days when compared to zero to one day, but they described a reduction in positivity rates after this period. Finally, nasopharyngeal washes typically yield higher viral titers than nasopharyngeal swabs [38], and the BD Veritor ^TM^ test was only indicated for use in nasal and nasopharyngeal swabs [20] and not oral pharyngeal swabs at the time of our study. Given the improved influenza viral yield from the use of NP/OP relative to NS swabs, future validation of the RIDT assay using NP/OP specimens could be warranted. Indeed, when compared to rRT-PCR undertaken on NP/OP swabs, the use of the RIDT on NS specimens resulted in our missing ~30% of true influenza cases. This was due in part to the reduced performance of the RIDT against rRT-PCR, and in part to the use of NS vs NP/OP swabs.

## Conclusion

In conclusion, the BD Veritor^TM^ System RIDT demonstrated a moderately high sensitivity and high specificity in NS specimens when used predominately in young children at pediatric clinical sites in Kenya. While they are not sufficient on their own for clinical decision-making related to influenza in these contexts, RIDTs may have a role in promoting sustainable and timely influenza surveillance, particularly in remote locations where transportation of specimens to laboratories for rRT-PCR testing is difficult.

## Abbreviations

BD: Becton Dickinson
CDC: Centers for Disease Control
CI: Confidence intervals
*C*_*T*_: threshold cycle
GA: Georgia
IHR: International Health Regulations
ILI: Influenza like illness
IRB: Institutional review board
KEMRI: Kenya Medical Research Institute
NC: North Carolina
NP: Nasopharyngeal
NPV: Negative predictive value
NS: Nasal Swab
OP: Oropharyngeal
PBIDS: Population Based Infectious Disease Surveillance
PPV: Positive predictive value
RIDT: Rapid influenza diagnostic test
RNP: Ribonucleoprotein
RRT-PCR: Real time reverse-transcription polymerase chain reaction
SARI: Severe acute respiratory illness
SERU: Scientific and ethics review unit
SQL: Structured query language
SSC: Scientific steering committee
USA: United States of America
VTM: Viral transport media
WA: Washington
